# Medical Liability in Cancer Care During COVID-19 Pandemic: Heroes or Guilty?

**DOI:** 10.3389/fpubh.2020.602988

**Published:** 2020-12-18

**Authors:** Rosario Barranco, Carlo Messina, Alessandro Bonsignore, Carlo Cattrini, Francesco Ventura

**Affiliations:** ^1^Department of Legal and Forensic Medicine, University of Genoa, Genoa, Italy; ^2^Department of Medical Oncology, Santa Chiara Hospital, Trento, Italy; ^3^Department of Internal Medicine and Medical Specialties (DIMI), School of Medicine, University of Genoa, Genoa, Italy

**Keywords:** COVID-19, SARS-CoV-2, medical liability, pandemic, cancers

## Abstract

**Background:** The COVID-19 outbreak rapidly became a public health emergency affecting particularly the frail category as cancer patients. This led oncologists to radical changes in patient management, facing the unprecedent issue whether treatments in oncology could be postponed without compromising their efficacy.

**Purpose:** To discuss legal implications in oncology practice during the COVID-19 pandemic.

**Perspective:** Treatment delay is not always feasible in oncology where the timing often plays a key role and may impact significantly in prognosis. During the COVID-19 pandemic, the oncologists were found between the anvil and the hammer, on the one hand the need to treat cancer patients aiming to improve clinical benefits, and on the other hand the goal to reduce the risk of COVID-19 infection avoiding or delaying immunosuppressive treatments and hospital exposure. Therefore, two rising scenarios with possible implications in both criminal and civil law are emerging. Firstly, oncologists may be “accused” of having delayed or omitted the diagnosis and/or treatments with consequent worsening of patients' outcome. Secondly, oncologists can be blamed for having exposed patients to hospital environment considered at risk for COVID-19 transmission.

**Conclusions:** During the COVID-19 pandemic, clinical decision making should be well-balanced through a careful examination between clinical performance status, age, comorbidities, aim of the treatment, and the potential risk of COVID-19 infection in order to avoid the risk of suboptimal cancer care with potential legal repercussion. Moreover, all cases should be discussed in the oncology team or in the tumor board in order to share the best strategy to adopt case by case.

## Introduction

The outbreak of coronavirus disease 2019 (COVID-19) became a public health emergency, since the World Health Organization declared the novel severe acute respiratory syndrome coronavirus-2 (SARS-CoV-2) a pandemic on 11th March 2020 (1). Although the severity of this disease and the risk of death seem to be associated with old age and preexisting comorbidities such as cardiovascular disease, diabetes, and chronic obstructive pulmonary disease, cancer patients and cancer survivors could represent additional high-risk categories due to anticancer agent-related immunosuppression (2). Although the additional attributable risk to cancer is still unknown, there are some evidences showing a significant risk of COVID-19 infection among cancer patients over the age of 60 and concomitant lung comorbidities (3). This led clinicians to radical changes in patient management having the hard task of restructuring health systems to effectively manage the pandemic and at the same time provide the continuum of care (4). Therefore, following the Chinese model, globally many institutions were forced to adopt emergency measures such as workforce redeployment and reduction in capacity of oncology unit members due to staff shortages and to promptly adopt containment measures such as cancellation of scheduled surgical procedures and switching standard follow-up visits into phone follow-up visits or using other means of telemedicine in order to reduce hospital exposure (5).

This article describes the challenges handled by oncologists in providing cancer treatment during the COVID-19 pandemic, in particular the difficult task of balancing the expected benefits of treatment vs. the risk of exposing patients to SARS-CoV-2 infection and potential complications. Delayed treatment delivery and changes in treatment regimens can have a potential detrimental effect on prognosis and may expose treating oncologists to legal action against them.

## Medico-Legal Implication in Cancer Care

This public health emergency forced clinicians to make difficult decisions concerning the timing of care (immediate vs. deferred) and to establish which treatments were essential for a relevant impact on prognosis (3). Therefore, during COVID-19 clinicians were called to find a compromising between the benefit achieved by immediate treatment and the possible odds of infection.

In this regard, oncologists were particularly under pressure given the growing concern for patients' vulnerability and often faced the unprecedent issue whether effective treatments could be postponed without compromising their efficacy (4).

Obviously, the delay of treatments is not always feasible in the oncology where often the timing of diagnosis and treatment may play a crucial role for the prognosis (6). Hence, in many cases the oncologists were found between the anvil and the hammer, on the one hand the need to treat cancer patients aiming to improve clinical benefits, and on the other hand the goal to reduce the risk of COVID-19 infection avoiding or deferring immunosuppressive treatments and hospital exposure.

Certainly, the pandemic has important medico-legal implications (7, 8). Unfortunately, despite the severity of the pandemic and the initial choral praise of the population for the utmost efforts of health personnel, this led to important repercussions in the field of legal medicine and numerous episodes of actions were undertaken against the legal liability of doctors (9). Therefore, several cases against medical malpractice or, more generally, regarding the responsibility of medical administrators for the inadequate measures of infectious risk control emerged, complaining on the drastic increase in deaths in nursing homes for elderly patients, but also against the inadequate medical care in non-COVID-19 emergencies (10).

Therefore, two rising scenarios with implications in both criminal and civil law are possible in oncology. Firstly, oncologists may be “accused” of having delayed or omitted the diagnosis and/or treatments with consequent worsening of patients' outcome. Secondly, oncologists can be blamed for having exposed patients to a hospital environment considered at risk for COVID-19 transmission. However, clinicians should be cautious and warned for forensic implications.

From a medico-legal point of view, in the first scenario the clinician may be “accused” of having delayed (or omitted) the diagnosis and/or treatment. The correlation of events between delay in diagnosis/treatment and disease progression could be investigated through a forensic study, analyzing tumor growth and progression over time with subsequent change in prognosis.

This change in prognosis and the reduction in life expectancy could be a relevant reason for medical malpractice.

On the other hand, oncologists can be liable for having exposed the patient (having to go to the hospital for treatment) to the infection. In these cases, it is very complex to distinguish whether COVID-19 infection occurred due to immune-suppression treatment related to any social contact inside and/or outside the hospital. Therefore, understanding whether the infection is related to the treatments or is independent is a complex task.

Only a close monitoring of all patients' contacts may give useful information for tracing those possibly responsible for the COVID-19 transmission.

Clinical decision making should be well-balanced through a careful examination between clinical performance status, age, comorbidities, aim of the treatment (cure vs. palliation), and the potential risk of COVID-19 infection in order to avoid the risk of suboptimal cancer care with potential legal repercussion (2).

Although avoiding or deferring effecting treatment in oncology during the COVID-19 pandemic is still a matter of debate (2, 11), in our opinion this concern involved only limited cases in daily clinical practice.

Immediate treatment should be promptly considered for those tumors at high risk of early mortality and highly sensible to chemotherapy (i.e., acute leukemia, aggressive lymphomas, metastatic germ cell tumors) where the cancer-related prognosis is poorer than COVID-19-related mortality. In the midst of the pandemic, an international survey among experts belonging to three cooperative groups (Italian germ cell tumors, European G3 domain, genitourinary medical oncologists of Canada) posed the question whether the delay of treatment would be acceptable for a highly curable cancer as germ cell tumors (GCT) (12). Although there was a large consensus among experts in treatment discontinuation or delay for COVID-19-positive patients, management strategies of COVID-19-free GCT patients remained intact reflecting the priority to guarantee a high standard of care for GCT patients, as shown by the low rate of elective surgical delay as well as the management of poor-risk patients (12). Moreover, an immediate local treatment should be always offered in patients with localized disease where surgery or radiotherapy may play a curative role (13, 14). Suboptimal delivery of radiotherapy or surgery has been demonstrated to compromise both local control and survival (13, 14). For example, delaying the initiation of adjuvant radiotherapy >8 weeks after surgery doubles the risk of local recurrence in patients with breast cancer (15). Similarly, delaying the initiation of surgery in patients with stage II or III colon cancer negatively impacted overall survival (14).

Therefore, many institutions showed that radiotherapy has been safely delivered during the COVID-19 pandemic especially when used with curative intent, and in some clinical scenarios it could replace surgery maintaining similar outcomes avoiding intensive care unit occupation (i.e., radical radiotherapy on the prostate instead of radical prostatectomy in high-risk localized prostate cancer, concomitant chemotherapy, and radiotherapy for cervical cancer instead of surgery). Furthermore, many centers increased the use of hypofractionated regimens, which minimize the number of visits to hospitals while also avoiding potentially detrimental delays in the delivery of cancer care (13).

However, treatment delay may be taken into account in tumors slowly progressing and low early cancer mortality (i.e., basal cell carcinomas or low-risk prostate cancer) where the lethality due to COVID-19 infection is likely to be higher than cancer-specific mortality. In these cases, it is likely that treatment delay does not change the prognosis. Moreover, in these circumstances standard follow-up should be replaced with telematic evaluations (6). The most difficult task of choice is limited to other neoplasms (i.e., bladder cancer, breast cancer, colorectal cancer, lung cancer, melanoma, etc.) in which a diagnosis (through screening) or punctual therapy could change the prognosis. An Italian survey conducted among members of Italian association of cancers and the Italian breast cancer study group showed some potentially alarming signals of undertreatment (16). In the neoadjuvant setting, fewer oncologists compared with those before the emergency adopted weekly paclitaxel (68.5 vs. 93.9%) and a dose of anthracycline-based chemotherapy. Similarly, in metastatic settings fewer oncologists compared with those before the emergency adopted weekly paclitaxel upfront for Her-2-positive disease (41.8 vs. 53.9%) or CDK 4/6 inhibitors for ER-positive HER2-negative metastatic breast cancers with less-aggressive features (55.8 vs. 80%) (16).

Similarly, delays in chemotherapy for colorectal cancer is associated with lower survival. Furthermore, there is a 16% increase in the risk of death for every month of delay in radiation therapy for patients with head and neck cancer (6, 17–19). Moreover, given the uncertainty of an interference between immune checkpoint inhibitors and Sars-COV-2 pathogenesis, a survey conducted among Italian physicians involved in the administration of immune checkpoint inhibitors in oncology explored their perception about SARS-CoV-2-related risks in patients with solid tumors receiving these therapies, and the attitudes toward their management during the COVID-19 outbreak (20). Almost 47% of oncologists supported the hypothesis of a synergism between the mechanism of action of immune checkpoint inhibitors and the pathogenesis of SARS-CoV-2 infections and were concerned about the potential higher risks of COVID-19-related complications in cancer patients. Nevertheless, it was reassuring that 97.1% of respondents would not deny immune checkpoint inhibitors as a treatment option at the time of the COVID-19 outbreak only based on the possible risks of infection by SARS-CoV-2, considering the lack of evidence of a detrimental effect of their administration (20).

In this context, the clinicians are at risk of important legal consequences. For example, in Italy the doctor risks a conviction for manslaughter or personal injury (or impairment of health) from a criminal law point of view, whereas from the civil law perspective, the clinician risks to compensate (through insurance) a large sum of money (compensation for damage).

In these cases, the costs of the medico-legal dispute can increase the insurance charges. Therefore, economic resources are allocated to compensation for damage and are subtracted from the resources destined to improve the health service for the needs of patients.

No judgments have been delivered in this area yet, so we do not know the jurisprudential orientation. In our opinion, the delay in diagnosis/therapy of neoplasms (with poor prognosis if not treated immediately) could be justified (or in part partially “forgiven”) in geographical areas with a very high incidence of infections (for example Lombardy), whereas an excessively prudent health attitude would not have enough justifications in areas with low incidence of COVID-19.

Furthermore, the reduction of the risk of transmission of SARS-CoV-2 through hospitals should be limited by an adequate triage and oncologists should provide complete information regarding drawbacks and benefits that are treatment-related as well as treatment plans which should be shared and accepted by the patients signing a written informed consent. Moreover, all cases should be discussed in the oncology team or in the tumor board in order to share the best strategy. At least, several efforts have been made by national and international scientific societies to offer guidelines for the delivery of anticancer treatment for standardized cancer care among different institutions, thus limiting the risk of medical malpractice and medico-legal implications (21).

## Conclusions

COVID-19 has overwhelmed the capacity of the health system. Postponing cancer treatment is associated with certain risks. The latter should be balanced by benefits yielded by anticancer agents, and clinical decision making should be discussed in the tumor board following international guidelines on the management of cancer patients' during the COVID-19 pandemic (21). The oncologists must do everything possible to avoid the risk of suboptimal care.

Abdul-Rahman Jazieh et al. (22) developed a detailed plan to help oncology services during a major coronavirus outbreak. The main objective was the prevention of new infections in the oncology service, managing currently infected patients and providing timely treatment of cancer for the entire patient population. The plan analyzed the management of infected patients, preventing new infections in patients or healthcare personnel, ensuring the continuity of cancer care, and incorporating measures to support these interventions up to the post-epidemic period. On the basis of this study, in our opinion patients should be divided into 3 general categories:

- Urgent: where surgical treatment, chemotherapy, or radiotherapy should not be postponed because of the high risk of worsening the prognosis.
- Intermediate: all cases should be discussed within the tumor board. Surgery may be rescheduled after a short delay, and the feasibility of chemotherapy and radiotherapy should be discussed case by case balancing risks and benefits.
- Postponable: the postponement of interventions does not change the prognosis. Therefore, if the risk of infection is high, we recommend postponing active treatment.

In any case, it is always necessary to test in-patients with nasopharyngeal swab at hospital admission and all out-patients before starting every cycle of systemic therapy. Similarly, healthcare personnel must be tested for SARS-CoV-2 periodically in order to avoid clusters of COVID-19 transmission within the Oncology Unit.

## Data Availability Statement

The original contributions presented in the study are included in the article/supplementary material, further inquiries can be directed to the corresponding author/s.

## Author Contributions

RB and FV: conceptualization. CM: methodology. CC: software. FV, AB, and CC: validation. RB and AB: formal analysis. RB: investigation, resources, and writing—original draft preparation. CM: data curation, writing—review and editing, and visualization. FV: supervision, project administration, and funding acquisition. All authors contributed to the article and approved the submitted version.

## Conflict of Interest

The authors declare that the research was conducted in the absence of any commercial or financial relationships that could be construed as a potential conflict of interest.
